# Methylene blue as a new signal tracer for nucleic acid-based lateral flow assay

**DOI:** 10.1038/s41598-025-19701-4

**Published:** 2025-10-14

**Authors:** Jurjaan Onayza Noim, Dhruvi Kakadiya, Stephanie Dang, Nabil Royez, Shruti Ahuja, Krishna Prasad Aryal, Siddharth Tallur, Dylan Ravindran Pillai, Richa Pandey

**Affiliations:** 1 Department of Biomedical Engineering, University of Calgary, 2500 University Dr NW, T2N 1N4 Calgary, AB Canada; 2 Department of Microbiology, Immunology, and Infectious Diseases, University of Calgary, Calgary, Canada; 3 Centre for Research in Nanotechnology & Science (CRNTS), Indian Institute of Technology (IIT) Bombay, Mumbai, India; 4 Department of Electrical Engineering, Indian Institute of Technology (IIT) Bombay, Mumbai, India; 5 Department of Pathology and Laboratory Medicine, University of Calgary, Calgary, Canada; 6 Hotchkiss Brain Institute, University of Calgary, 3330 Hospital Dr NW, Calgary, AB T2N 4N Canada

**Keywords:** Lateral flow assay, Nucleic acid detection, Methylene blue, Quantitative DNA detection, Electrochemical biosensor, Diagnosis, Biomedical engineering, Sensors and biosensors

## Abstract

We report the development of MebiQue-LFA, a nucleic acid-integrated lateral flow assay that utilizes methylene blue (MB) as a new colorimetric signal tracer for detecting nucleic acids. MebiQue-LFA advances LFA technology by enabling visual detection via blue dye and enhancing sensitivity through integrated electrochemical transduction on the same platform. The electrochemical readout provides a sensitive and quantifiable alternative when visual detection is limited at low analyte concentrations. To demonstrate dual-mode detection, the assay was designed to target a model single-stranded DNA sequence, achieving a detection limit (DL) of 0.62 fM and 43 fM (~ 100 copies/µL) in buffer and contrived diluted (50%) blood respectively, with a dynamic range spanning 1 pM to 1 µM. The platform exhibited high specificity, discriminating single-base mismatches and non-complementary sequences. Furthermore, MebiQue-LFA demonstrated robust performance in complex biological matrices, including plasma and serum, achieving a DL of 16 pM (~ 10^5^ copies/µL). The platform is cost-effective (~ 3 CAD per test), rapid (15 min), stable in different storage conditions and compatible with mobile readout platforms, highlighting its potential for decentralized and point-of-care nucleic acid diagnostics.

**Supplementary Information:**

The online version contains supplementary material available at 10.1038/s41598-025-19701-4.

## **Introduction**

Signal tracers are detectable markers, such as nanoparticles, dyes, or enzymes, that are used in diagnostic assays to indicate the presence of a target analyte. They are critical in enabling sensitive, accurate, and often quantitative detection^1^. Methylene blue (3,7-bis(dimethylamino)phenazathionium chloride, MB) is a blue-colored dye with exceptional electroactivity utilized as a signal tracer in electrochemical biosensing assays^2,3^. Its redox-mediating properties make it suitable for nucleic acid bioreceptor modification, facilitating fast electron transfer at a suitable applied potential difference between electrodes^4–6^. In specific applications, such as DNA damage detection, methylene blue is utilized for its ability to intercalate into the double helix structure of dsDNA, providing a basis for electrochemical sensing^7^. Many electrochemical DNA biosensors exploit the conjugation chemistry of MB with the 3’ or 5’ end of the ssDNA or dsDNA for an enhanced signal-to-noise ratio^8,9^.

With its multifunctionality in producing colored and electrochemical signals, MB promises to be a good alternative for many of the “gold standard” signal tracers used in lateral flow assays (LFAs). Other molecular dyes like Prussian blue (PB) and crystal violet (CrV) have been used in LFAs^10^, but their limitations include instability to environmental conditions, complex modification, and lack of dual functionality^11–13^. Colored dye, CrV, is commonly used for biological staining, particularly in Gram staining of prokaryotic cells, where it binds to peptidoglycans through non-covalent interactions^14^. CrV is also utilized in commercial applications such as textile dyeing and protein staining in SDS-PAGE and zymograms^15^. However, its functionality in an LFA is limited^16^. CrV can only bind to proteins and does not offer the dual readout capabilities (colorimetric and electrochemical) essential for more versatile and sensitive diagnostic applications. In contrast, MB offers significant advantages as a signal reporter in LFAs. MB’s dual-modality capabilities allow for colorimetric and electrochemical detection, enabling precise quantification even at low concentrations where CrV and PB may falter. Its stability, combined with the ability to deliver both visual and electrochemical readouts, makes MB a more versatile and practical choice for LFAs, particularly in demanding diagnostic environments^4^. A dual signal (fluorometry and colorimetry) use of MB for detecting *E.coli* cells on an LFA has been demonstrated. However, the assay requires complex chemicals such as Zirconia-based organic framework and Aluminum ions for the signal change^17^. However, MBs’ application for a Nucleic Acid-Based Lateral flow Assay (NALFA) has not been demonstrated for rapid and point-of-care diagnostics applications.

The core concept of using a single label to enable optical and electrochemical transduction in lateral flow systems has been explored in existing literature^18,19^. A promising approach is utilizing a single label by integrating electrochemical detection with LFAs, forming electrochemical lateral flow assays (e-LFAs)^20^. These offer a broader detection range, high reproducibility, and potential for real-time measurement. This integration provides a comprehensive approach, leveraging the strengths of both techniques for improved analytical capabilities in various sensing applications. Miglione et al. demonstrate that the electrochemical lateral flow assay (eLFA) offers a decentralized platform, combining AuNP-based LFA with a smartphone-powered portable potentiostat for electrochemical detection. This enables a user-friendly test by eliminating the necessity of expensive laboratory equipment and skilled personnel to administer the test. This integration enhances analytical capabilities without adding complexity, providing a frugal alternative for point-of-care testing with minimal experimental tasks. Recent eLFA advancement, by Petruzzi *et al. and* Deeni et al.., such as C-reactive protein (CRP) quantification in saliva, offering up to 50 times higher sensitivity than traditional methods^21^. Deeni et al. further enhanced eLFA sensitivity, achieving up to 50 times higher sensitivity than conventional methods, thus meeting medical-device standards for diagnostic accuracy^22^. These advancements highlight the growing significance of eLFA in point-of-care testing and pave the way for developing portable and wearable diagnostic sensors with enhanced diagnostic performance. AuNPs offer visible signals, ease of synthesis, stability, and simplicity in handling; however, they cannot provide quantitative results^23,24^. The use of colloidal AuNPs in LFAs presents advantages and limitations. AuNPs exhibit low optical sensitivity, affecting the limit of detection. While cost-effective and commercially available, their qualitative interpretation restricts applicability in disease screening, where more quantitative results are required^24,25^. Alternative labels like latex, carbon, and fluorescent nanoparticles offer improved sensitivity and multiplexing capabilities^26,27^. However, fluorescent nanoparticles may introduce background noise (nonspecific fluorescent signal), requiring signal-to-noise ratio enhancement. Quantitative LFAs using optical, electrical, or magnetic readers address the qualitative nature of many tests, but the cost and additional steps limit their use^28^.

In our approach, we have employed Methylene Blue as a signal tracer for the colorimetric and electrochemical assay, leveraging its dual modality for detecting nucleic acids on a single device without any amplification step. While Methylene Blue’s role in electrochemical biosensors is well established^29,30^our research highlights its mechanistic use as a colored dye, as an alternative to nanoparticles in nucleic acid-based lateral flow assays. Methylene blue’s dual-modality signal tracing capability offers advantages in nucleic acid detection. The electrochemical readout provides high sensitivity at lower analyte concentrations, where equipment-free visual detection is limited, ensuring precise quantification even when the visual signal fades. Separating the biorecognition on the lateral flow strip and detection on the reagent less electrochemical chip events minimizes biofouling on the electrochemical detection, enhancing performance in complex biological samples. To our knowledge, a simplified nucleic acid-based LFA utilizing only MB as a single label for dual-modality has not been demonstrated.

This research presents the following advancements in the field of colorimetric signal tracers, NALFAs and e-LFAs:

1. This research introduces the Methylene Blue Integrated Quantitative Electrochemical Lateral Flow Assay (MebiQue-LFA), a new rationally designed fully reagent-integrated nucleic acid lateral flow assay (NALFA) platform that uniquely combines colorimetric and electrochemical signal outputs for robust, dual-mode detection of nucleic acids. As a compelling proof of concept, the MebiQue-LFA was engineered to detect single-stranded (ss) DNA sequences, demonstrating reliable mechanistic validation.
2. For the first time, MB is employed as a colorimetric signal tracer on a NALFA platform. The study systematically explores the MB’s signal-generation mechanism, marking a novel advancement in LFA design and functionality.
3. The MebiQue-LFA assay design alleviates biofouling (a common challenge in biosensors) via multimodal technology integrations without requiring an anti-biofouling coating or reagents. Our new assay design separates biorecognition and sensing into two steps, hence eliminating the need for any anti-biofouling layer on the bio-receptor modification-free, low-cost PCB electrodes.
4. The platform achieves sensitive detection, reaching 1 pM in diluted blood without any amplification step, and maintains high specificity, even discriminating against single-nucleotide mismatches. Furthermore, it demonstrates consistent performance across untreated biological matrices—plasma, serum, and whole blood.
5. MebiQue-LFA is a cost-effective, highly sensitive, field-deployable solution that delivers visual and quantitative readouts in under 15 min. Its performance is stable for upto 10 days in different storage conditions. Its user-friendly interface, minimal equipment requirement, and real-time mobile connectivity make it an ideal candidate for decentralized diagnostics in resource-limited or point-of-care settings.

## Results

### MebiQue-LFA assembly and optimization

**Fig. 1:**
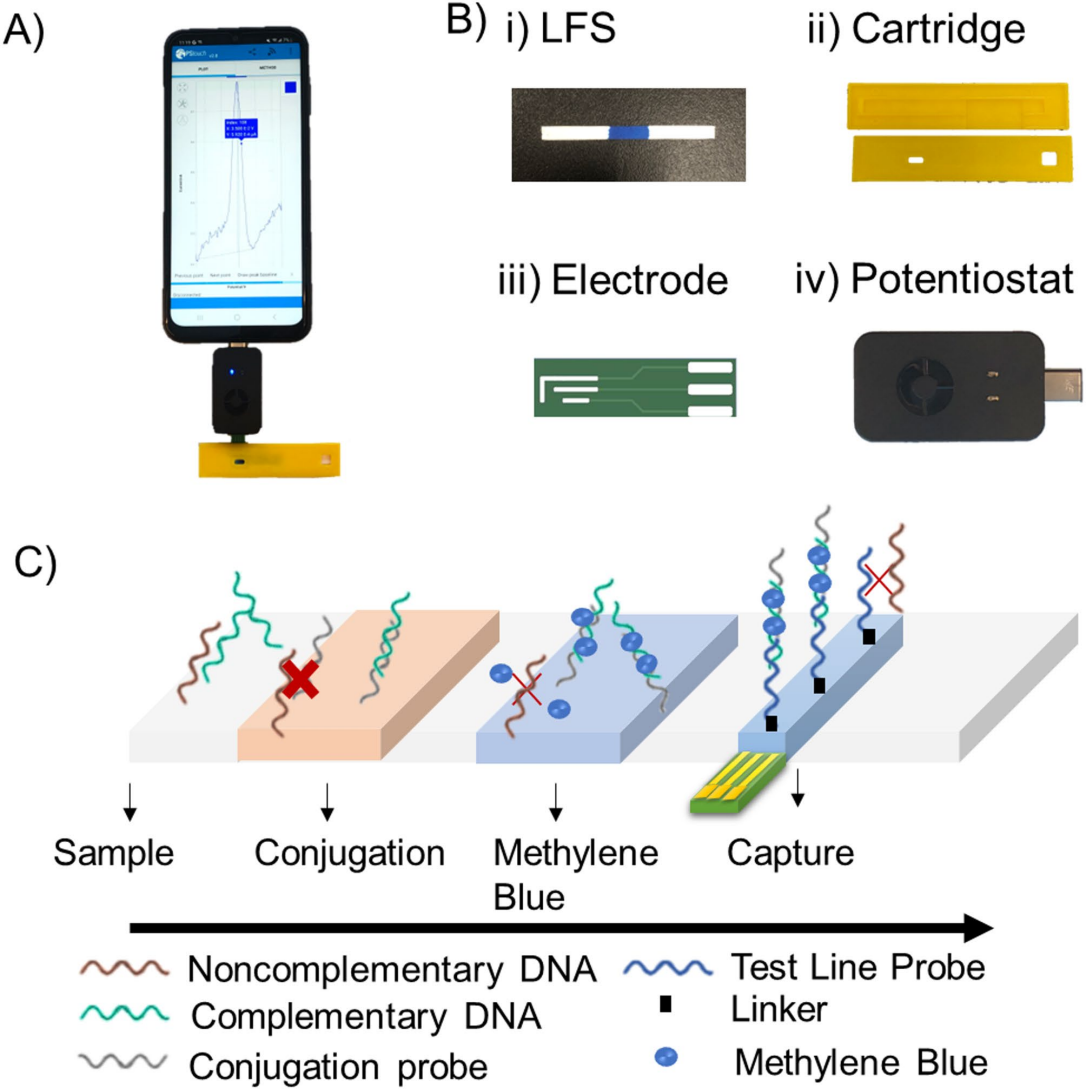
(**A**) MebiQue-LFA: **Me**thylene **b**lue **i**ntegrated **Qu**antitative **e**lectrochemical-**L**ateral **F**low **A**ssay. Schematic overview of the fully integrated device and its connectivity to mobile devices for real-time data transmission. (**B**) Individual components of the assay: reagent-integrated LFS, cartridge, electrode, and potentiostat for targeted DNA analysis. (**C**) Schematic depiction of assay mechanism: visual representation of the interaction between target DNA and complementary conjugate pad, forming a duplex, followed by methylene blue intercalation and capture by a dedicated probe in the test line region.

The multimodal DNA detection platform provides a fully integrated and point-of-care solution to DNA detection using eLFA [Figure 1A]. The fully integrated MebiQue-LFA comprises four integral components: a reagent-integrated lateral flow strip (LFS), cartridge, electrode, and Potentiostat [Figure 1B]. Positioned on the bottom casing of the cartridge, the assay interfaces with an electrode beneath the LFS, connected to an electrochemical reader, ultimately transmitting electrochemical data to a mobile phone via a USB-C connector.

The nucleic acid-based assay on the LFS is designed to detect a ss oligonucleotide as a proof of concept. The LFA encompasses a sample pad where the analyte sample with a specific target sequence is placed in low volumes (minimum 30 µL). The analyte progresses through a conjugation pad, which houses sequences complementary to the target sequences. The innovative aspect of our assay lies in utilizing a dye pad containing methylene blue, marking a pioneering use of this colored and redox molecule in a combined colorimetric-electrochemical assay. The conjugated target interacts with methylene blue through multiple mechanisms: groove-binding, electrostatic interaction, and predominant intercalation, a process where methylene blue inserts itself between adjacent base pairs of the target DNA or oligo sequences^6,7^. This MB-DNA complex is subsequently captured by a specific capture probe located in the test line region [Figure 1C. The assay design merely requires knowledge of any particular sequence of varied lengths. After obtaining this knowledge, a complementary conjugate pad and capture (test line) DNA are designed and tested for their interaction using gel electrophoresis (Figure S1). The binding of target DNA to conjugate pad DNA leads to a noticeable shift in mobility during agarose gel electrophoresis. This shift becomes more pronounced due to further binding with test line DNA, resulting in a discernible pattern observed on the gel. The final step involves electrochemical measurements facilitated by the electrode, providing a quantitative assessment of the assay performance by a redox reaction: MB^3+^ + e^-^ $\:\leftrightarrow\:\:$MB^2+^.^31^

In this study, a cost-effective printed circuit board (PCB) electrode was designed and tested for its electrochemical performance with common redox active solutions using cyclic voltammetry and square wave voltammetry (Figure S2). The electrode demonstrated comparable performance to existing PCB electrodes^32^. This integrated approach represents a significant advancement in nucleic acid-based assays. This unique use of methylene blue as a signal tracer enables us to harness all the advantages of eLFA. Methylene blue is a dual-function molecule, participating in the assay’s colorimetric and electrochemical aspects. Its ability to interact with DNA or oligo sequences enhances the assay’s sensitivity and ensures precise detection of target nucleic acid sequences in electrochemistry^33–36^. The redox activity of methylene blue further enables quantitative measurements, providing a comprehensive assessment of the quantity of DNA in the sample. This dual-mode assay contributes to its effectiveness in nucleic acid-based diagnostics, making it a significant advancement in the eLFA development and validation.

**Fig. 2:**
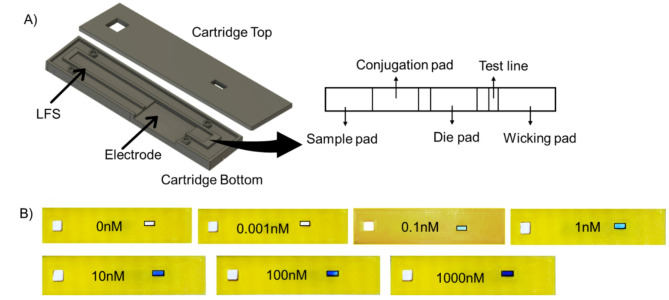
(**A**) CAD illustration of the cartridge design portrayed using Fusion360 CAD modeling software. The electrode and LFS placement on the bottom casing are strategically showcased, emphasizing their precise positioning for optimal functionality. The upper casing contains openings allowing the analyte to be introduced to the strip and observe the test line. A graphic overview of the LFS, illustrating the individual pad’s location. (**B**) Sensitivity test of the LFS, showing performance across the comprehensive range of target concentrations from 0 nM to 1000 nM, and a gradient of blue color can be seen over these concentrations.

The cartridge where our assay resides consists of (1) a top casing and (2) a bottom casing [Figure 2A]. The bottom casing, measuring 7.2 cm in length and 1.7 cm in width, features a central slot (6 cm x 0.5 cm) tailored to accommodate the LFS^37,38^. The dimensions of our developed assays, measuring 6 cm x 0.5 cm (length x width), differ slightly from the commonly observed LFS dimensions in commercial rapid diagnostic tests (RDTs), which are typically 4 cm x 0.4 cm (length x width). This difference in size is due to the placement of an additional dye pad requirement for our assay. This variance in size may have implications for the performance and utility of our assays compared to standard RDTs. The larger dimensions could offer advantages such as increased surface area for sample interaction, potentially enhancing sensitivity or accommodating larger quantities of analyte. Additionally, this casing includes a dedicated section for the electrode, allowing precise alignment with the position of the test line. The top casing, mirroring the dimensions of the bottom casing, distinguishes itself with two strategic openings. The first facilitates the introduction of the target analyte or sample (0.5 cm x 0.5 cm), while the second serves as a viewing window (0.5 cm x 0.2 cm) for monitoring the test line.

The width of the LFS was optimized [Figure S3] to achieve consistent sample flow, accurate timing of reactions, increased sensitivity, and prevention of potential over-saturation challenges. The different widths affect how fast the sample moves and how strong the signal appears on the test line^1,39^. By adjusting these sizes, we ensured that the test worked well by finding the right mix of sample interaction, reaction speed, and sensitivity while avoiding any issues that may be caused by the use of too much liquid^1^. Visual observations revealed that the analyte solution did not reach the test line for widths of 2 cm and 2.5 cm even after waiting for 45 min, while quicker fluid flow was observed for widths of 0.5 cm and 1 cm (Figure S3). The 0.5 cm width LFS exhibited superior performance, demonstrating the fastest wicking time (16 s) compared to other widths. Hence, 0.5 cm was selected as the optimum width for our LFS development.

Subsequently, a study was conducted on LFS with a 0.5 cm width to determine the optimum analyte volume [Figure S4]. This investigation aimed to assess how the assay would perform with low-volume analytes. Visual inspection revealed that a volume of 15 µL did not reach the test line even after 10 min. Conversely, higher volumes, such as 60 µL and 75 µL, led to faster fluid flow (117 s and 32 s, respectively) but resulted in excessive paper saturation. Volumes of 30 µL and 45 µL demonstrated optimal performance, reaching the test line with the appropriate saturation level (device integrity and no fluid leakage) in 10.45 min and 2.40 min, respectively. 30 µL volume was found optimal as the LFS remained intact and no residual fluid leaked from the strip after the sample front reached the distal end. Post-assay examination involved lifting the strip to assess structural integrity and detect any remaining solution around or beneath the device. This approach ensured reliable fluid migration and assay performance. The optimum time for a lateral flow assay typically ranges from 10 min to 1 h^40^.

The graphical representation [Figure S4] further confirmed that, despite taking more time (10.45 min), 30 mL exhibited performance optimum for a lateral flow assay. This choice was substantiated by its ability to reach the test line with optimal saturation, as demonstrated by a steady progression in the graph. The 30 mL volume avoided issues such as insufficient fluid reaching the test line (as observed with 15 mL) and steered clear of excessive saturation, which occurred at higher volumes (60 mL and 75 mL). The preference for 30 ml volume is also supported by the lower error bars observed in the graphs.

### Analytical performance validation of MebiQue-LFA

**Fig. 3:**
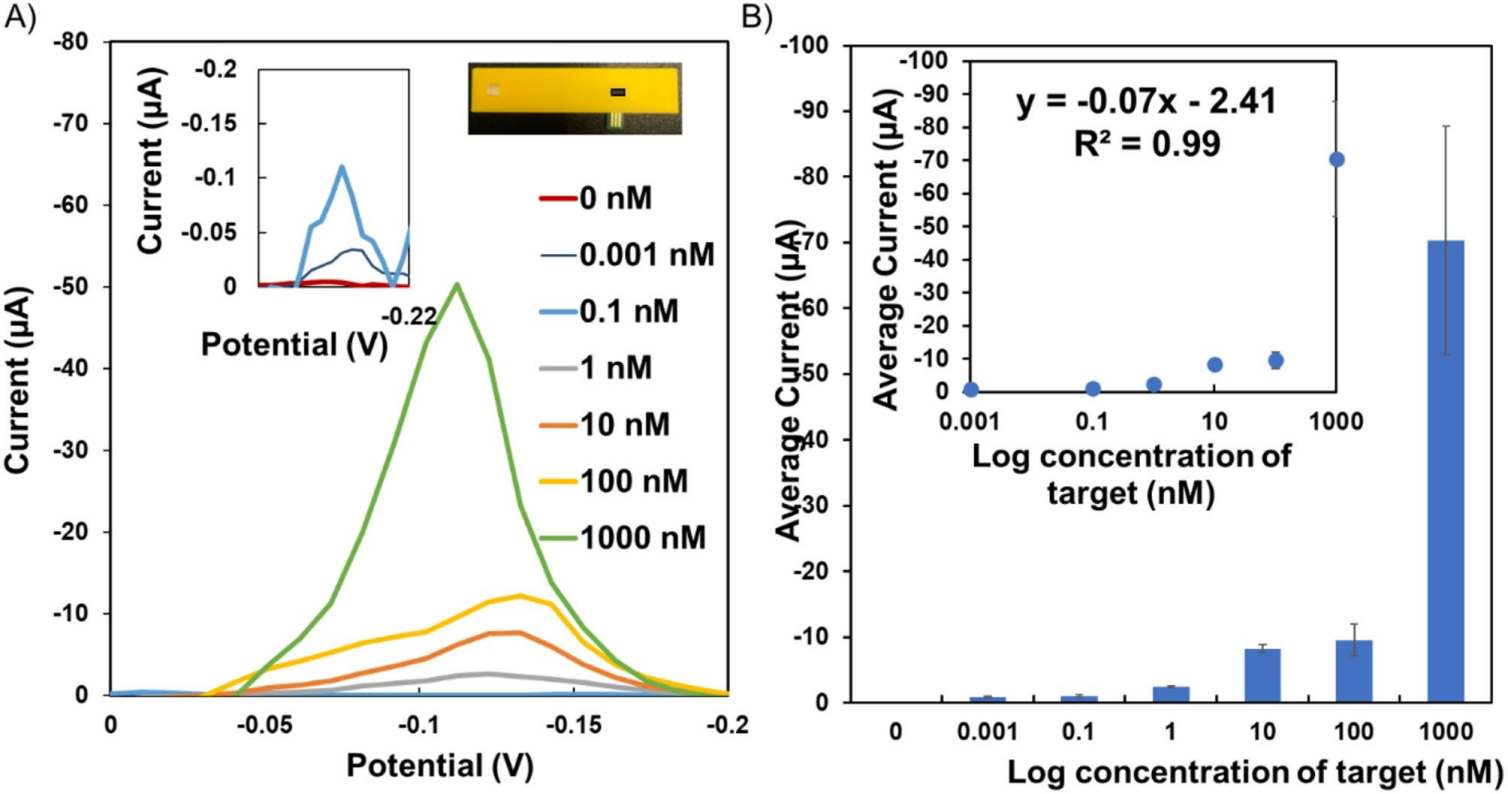
(**A**) Square wave voltammetry (SWV) measurements of the assay across a spectrum of concentrations (0 nM, 0.001nM, 0.1 nM, 1 nM, 10 nM, 100 nM, 1000 nM). Each concentration is mapped on the potential vs. current graph, showcasing distinct peaks corresponding to the electrochemical responses. Inset: SWV measurements of the assay for 0 and 0.1 nM. (**B**) Average current vs. log concentration plot. Each concentration point is plotted against its corresponding average current, and error bars represent the standard deviation of three electrodes.

The analytical performance validation study of the MebiQue-LFA platform comprised the interrogation of the colorimetric and electrochemical outcomes. An analytical sensitivity assessment of the MebiQue-LFA, was performed through colorimetry for different concentrations (0 nM, 0.001 nM, 0.1 nM, 1 nM, 10 nM, 100 nM and 1000 nM) of the target analytes [Figure 2B]. A distinct gradient of blue color was observed for varying concentrations of the target analyte, indicating the direct correlation between the concentration of the analyte and the color intensity in the colorimetric assay. Mechanistically, this color change is likely attributed to the interaction between the target-conjugation duplex and methylene blue, forming a binding complex. As the concentration of the target analyte increases, more target probe-conjugation pad probe duplex is formed, leading to a more pronounced and noticeable blue color.

**Fig. 4:**
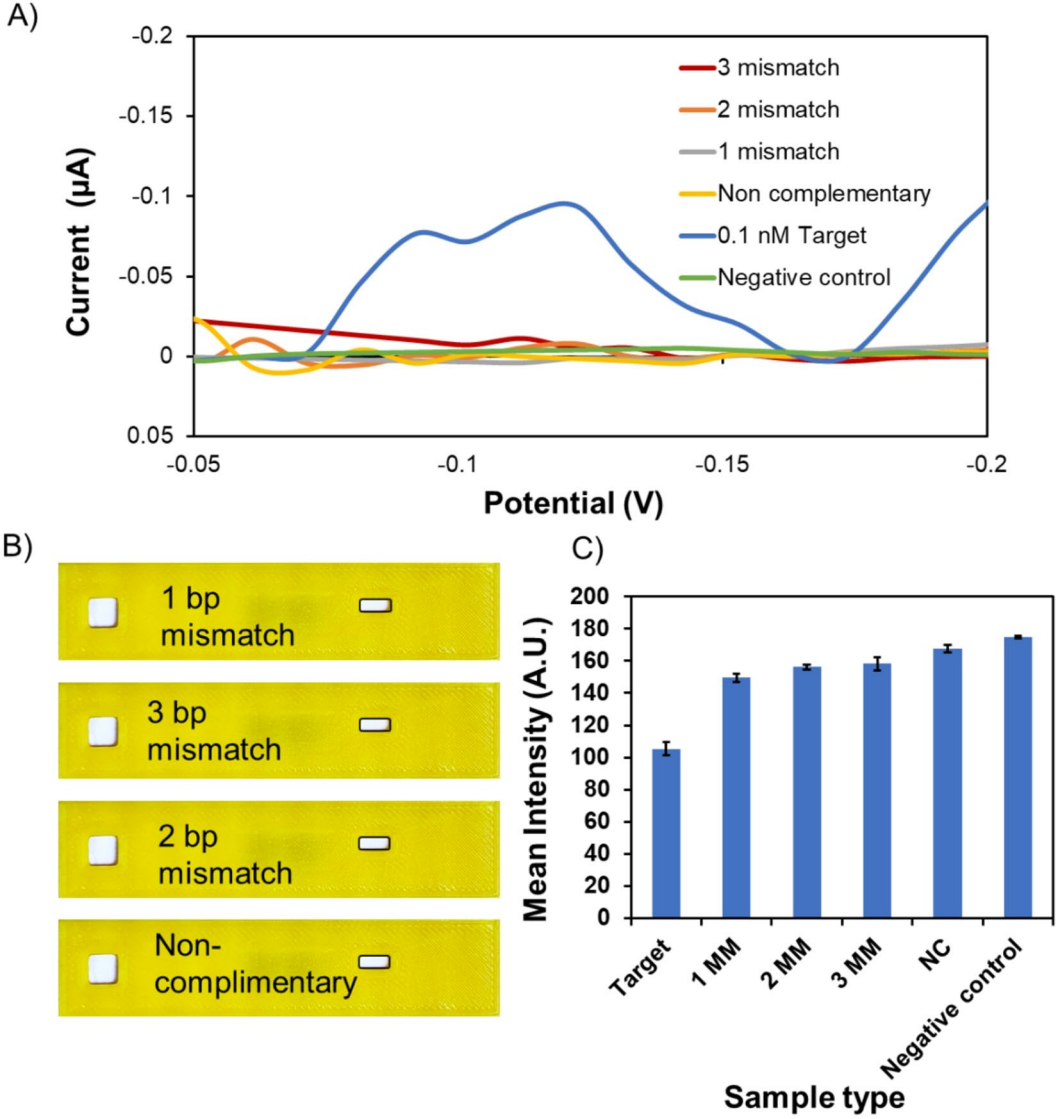
(**A**) Comprehensive analysis of the specificity of the assay using SWV. The graph illustrates the average current vs. potential for 1 µM of mismatches 1 (1 MM), 2 (2 MM), and 3 (3 MM) bp, non-complementary (NC) sequence compared with the 0.1 nM target concentration, and a negative control (0 target concentration). (**B**) Visual representation of the colorimetric lateral flow strip’s performance after specificity testing, focusing on mismatches at positions 1, 2, and 3 and a non-complementary sequence. (**C**) ImageJ analysis of the white light intensity of the test region. All the tests were performed for *n* = 3 devices.

We obtained a visible color change at 100 pM concentrations for the colorimetric outcome. The colorimetric output was affected by the proximity of concentration values, particularly between 10 nM and 100 nM. Despite challenges in achieving clear colorimetric differentiation, especially at lower concentrations (0, 0.001 and 0.1 nM), integrating electrochemical measurements proved highly effective in resolving these issues. The electroactive nature of methylene blue, harnessed for electrochemical measurements (Fig. 3A), significantly contributed to the precision of the assay in discriminating between these closely spaced (10 nM and 100 nM) concentrations. Figure 3B depicts the remarkable ability of the assay to detect 1 pM (~ 6 × 10^5^ copies/µL), experimentally. The detection limit calculated by considering the background signal and its standard deviation is 0.6 fM (~ 1 × 10^2^ copies/µL) without any amplification step. It has been shown that this can be further improved to as low as 1–10 copies/µL with an amplification step such as recombinase-aided amplification or isothermal amplification^41,42^. A specificity test was conducted to deepen our understanding of the selectivity of the assay to the target sequence and specific target-conjugation pad duplex formation [Figure 4A]. A negative control with the buffer alone and no target analyte is a crucial baseline reference for assessing specificity. The absence of colorimetric signals and electrochemical redox activity in this control establishes a clear indication of specificity in the absence of the target. We observed a smaller current peak for > 1000 times high concentrations (1µM) of 1–3 bp mismatches (5 ± 3 nA) signals. When compared with the 0.1 nM (0.1 ± 0.002 µA) target electrochemical signal, this was significantly less (20-fold) and reached the lower limit of sensitivity of our potentiostat (nano-amps). The visual observations of the assay in Fig. 4B demonstrated the absence of both colorimetric signals and electrochemical redox activity for these targets, highlighting the robust specificity of the assay. Figure 4C presents the white light intensity analysis of the lateral flow strips corresponding to the experimental conditions shown in Fig. 4A and B. The negative control exhibited the highest white light intensity (175 ± 85 A.U.), with a decrease observed as the target reached the lowest (105 ± 4 A.U.) at 0.1 nM target concentrations, where the test region appeared faint blue (Fig. 2B). Samples containing single, double, and triple bp mismatches (1MM, 2MM, 3MM), as well as the non-complementary (NC) sequence, displayed.

a white light intensity similar to non-complementary sample (149 ± 2 to 167 ± 2 A.U.), reflecting reduced hybridization efficiency, ranging from the strongest hybridization in the 1MM sample to the weakest in the non-complementary control. Notably, the white light intensities of all interferent samples remained close to that of the negative control, indicating minimal methylene blue signal development and thereby confirming the assay’s ability to distinguish even low concentrations of complementary target from mismatched or non-specific sequences. This specificity, even in closely related sequences, underscores the assay’s resilience against challenges in discerning target sequences. Furthermore, Fig. 4B and C findings corroborate with the baseline in Fig. 4A. Together, these experiments showcase target sequences’ reliable and specific discernment amidst various challenges using these assays, affirming their practical utility in specific nucleic acid detection applications.

Another specificity study was performed to determine the impact on sensitivity due to dsDNA target sequences in comparison with the ssDNA target sequences, as well as the need for conjugation pad (CP) sequences on assay performance. Figure S5 demonstrates that in the absence of conjugation pad sequence, there is no noticeable color change since the target sequence is an ssDNA that can interact with MB predominantly through G bases electrostatically as opposed to intercalative binding, groove (or surface) binding and electrostatic binding for ds-DNA^43,44^. The interaction with ssDNA overall is weaker, given that binding for ds-DNA there are 3 forms of interaction between MB-DNA (intercalation, groove binding, and electrostatic), whereas for ss-DNA, the MB-DNA interaction is only via electrostatic binding^45^. This study also validates the influence of the conjugation pad on the overall performance of the assay. The lack of colorimetric response in both the test and control experiments underscores the pivotal role of the conjugation pad in facilitating interaction between the target analyte and methylene blue, an interaction integral to the overall assay mechanism and functionality. Furthermore, the MB interacts with dsDNA (target- CP) via intercalation and/or groove binding.

A UV-Vis spectroscopy study was performed to elucidate the type of interaction between the assay’s MB and sequences (target, conjugate probe, and nonspecific mismatches). Figure S6 shows the MB absorbance maximum at 667 nm, which systematically decreases upon DNA addition. The simultaneous hypochromic effect and red shift are characteristic signatures of intercalative binding, indicating MB insertion between base pairs rather than external association^44–46^.

**Fig. 5:**
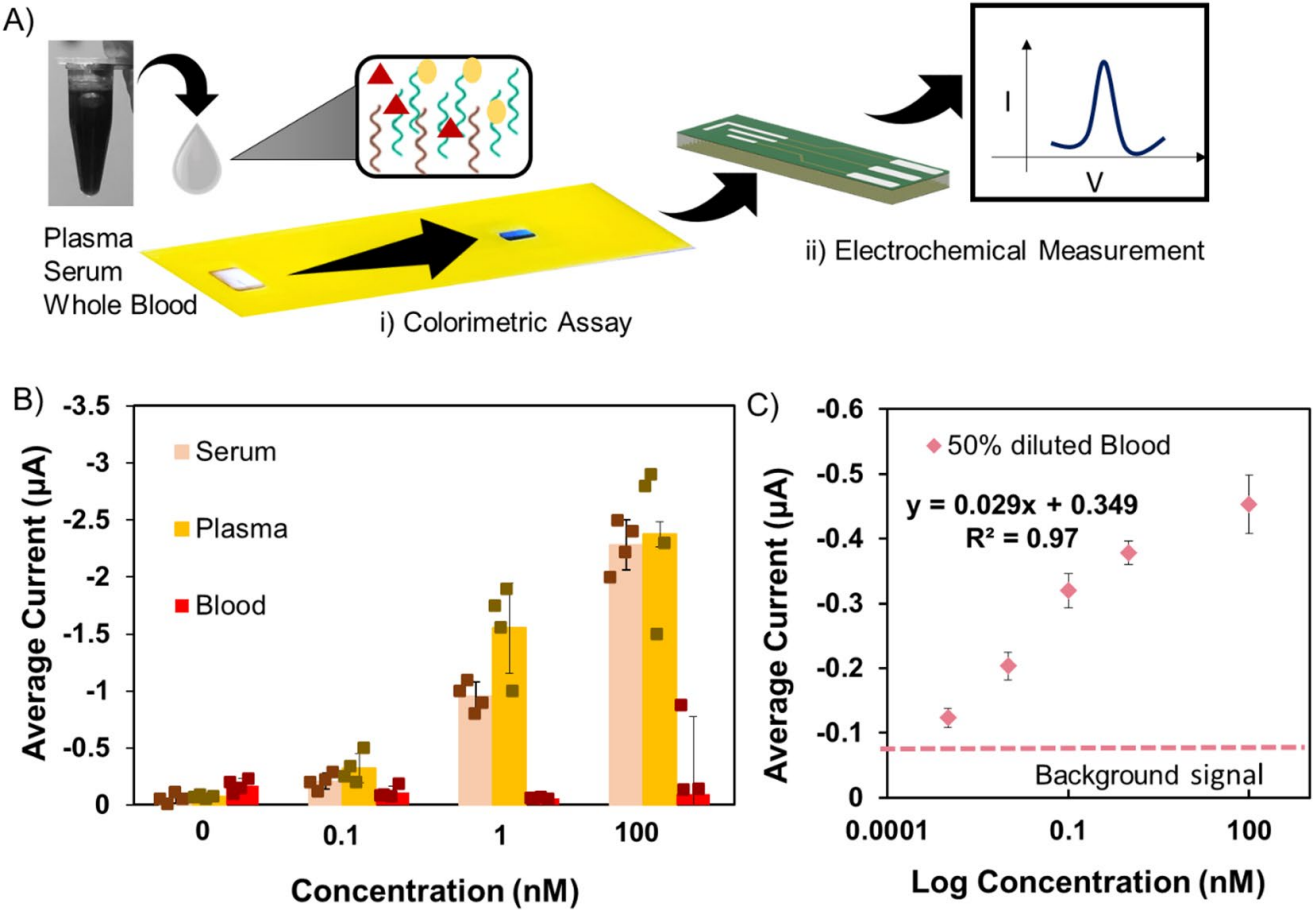
(**A**) Process flow of validating the MebiQue-LFA using biological samples. (**B**) Bar chart illustrating the electrochemical measurement of the MebiQue-LFA in different biological fluids: serum, plasma, whole blood. (**C**) Calibration curve for detecting ss DNA in 50% diluted blood. The background signal represents 3×standard deviations of the blank (50% diluted blood). Four and Six devices were tested for each measurement in 4B and 4 C respectively.

While G electrostatic quenching contributes to absorbance reduction, the differential binding between sequences with identical G content (TP, 1MM, 2MM at 26%) versus CP (11% G) demonstrates sequence-dependent intercalation preferences. Enhanced binding upon TP-CP duplex formation reflects cooperative intercalation effects in structured DNA. AA-TT base pairs, having lower stacking energies than GC pairs, provide thermodynamically favorable intercalation sites with reduced energy barriers for base unstacking^33^. Once intercalated, MB is stabilized through π-π stacking with nucleotide bases and dipole-dipole interactions with the DNA backbone, manifesting as the observed spectral perturbations^44–46^.

The stability test, as illustrated in Figure S7, evaluated the assay’s performance over 10 days under two different storage conditions: ambient room temperature and refrigerated storage at 4$\:℃$. The results demonstrated that the assay exhibits consistent performance regardless of the storage conditions, with minimal variation observed between the two environments (Table S3). The Coefficient of Variation % of 11.2% (Day-0) for the intra-day and 26.8% for the inter-day (Day- 10) variations demonstrate the optimal precision and stability of the MebiQue-LFA.

### Performance validation in contrived biological samples

For a comprehensive understanding of the practical utility of the assay in complex biological matrices, we conducted tests with contrived biological samples, seeking to elucidate its performance in real-world scenarios. This involved testing the assay with specific target sequences (0 nM to 100 nM) spiked in human plasma, serum, and whole blood (native and diluted). This was followed by a systematic evaluation of the assay using both qualitative colorimetry and quantitative electrochemical measurements [Figures 5A]. Notably, the observed outcomes varied across the tested fluids. Both serum (Figure S8A) and plasma (Figure S8B) demonstrated better performance, distinguishing between most concentrations colorimetrically. Electrochemically, we were able to obtain a distinguishable signal for 0.1 nM (*p* < 0.05) in both serum and plasma (Fig. 5B). In whole blood, the colorimetric and electrochemical performance deteriorated significantly, with the average current difference between 0 nM (no target) and 100 nM (highest) target analyte observed to be very low (0.09 ± 0.19 µA). Furthermore, some devices exhibited the presence of methylene blue in the test line, while others did not, indicating inconsistencies [Figure S8C]. This discrepancy suggests challenges in achieving reliable and consistent results when dealing with whole blood samples, plausibly due to interfering species impeding the overall assay’s performance. To further enhance the performance of MebiQue-LFA in blood, we tested our assay with various dilutions (10%, 30%, and 50%) of whole blood spiked with DNA concentrations of 0 nM and 100 nM [Figure S9]. The background signal for the native and diluted blood with no target analyte was very similar (0.15 ± 0.01 µA), demonstrating that there will always be a small current associated with the LFS test line interaction. The poor performance of the 10% and 30% diluted blood indicates the target loss due to over-dilution. The quantitative data aligns with our visual observations, affirming that 10% and 30% dilutions show low assay reliability in detecting target analytes in a blood matrix. Experimental detection of 1 pM target analyte in 50% diluted blood (Fig. 5C) showed a significant difference from the background average current signal (*p* < 0.05), surpassing the performance of native blood and the other two dilutions. We also achieved a calculated detection limit (43 fM) similar to the spiked buffer study.

## Discussion

This study demonstrates the use of methylene blue as a signal tracer and dye for the colorimetric lateral flow assay, offering advantages in nucleic acid detection over traditional nanoparticle-based tracers such as AuNPs. At lower analyte concentrations, where visual detection becomes challenging, methylene blue’s electrochemical readout ensures highly sensitive and precise quantification. This dual approach separates biorecognition and detection events, reducing biofouling on electrochemical chips, thereby enhancing performance in biological samples. The LFS fabrication and assay integration is simple with benchtop tools and methods. The recurring cost of the disposable device (LFS, cartridge, and the PCB electrode) was calculated to be CAD $3, with PCB electrode cost used in this calculation amounts to be the largest (~ CAD $2) due to low-volume production, which can be further reduced significantly by manufacturing larger quantities. The reader we used costs approximately $1,500, while a more basic reader can be obtained for less than $100^47^. The reader is a high-end commercial potentiostat with many functionalities that may not be required for this application. Further, in-house electronics development can make this platform fully automated and mobile device-free. The platform achieved a detection limit of 0.6 fM without amplification and with specificity against base-pair mismatches and noncomplementary targets. A dynamic range of 0.001–1000 nM was achieved in buffer-spiked target analytes. Further investigations with three biological matrices delineate similar performances in contrived serum and plasma at concentrations of 16 pM target analyte and above. While the whole blood didn’t achieve good performance, after diluting the spiked blood to 50% we were able to retrieve a similar LOD (43 fM) as the spiked buffer. Hence, this novel approach combines qualitative colorimetric assay with quantitative electrochemical sensing for pathogenic DNA detection in various biological samples. While the platform successfully alleviates some of the challenges of diagnostic assay performance in difficult-to-work-with biological samples, more studies need to be done to completely optimize the system performance with undiluted whole blood to avoid any sample pre-processing. While variations in sample color may potentially affect the visual intensity of the colorimetric signal, such differences are not expected to influence the electrochemical measurements significantly. In this study, we evaluated the assay performance across three biologically relevant matrices with distinct optical properties—serum (pale yellow), plasma (darker yellow), and whole blood (red-pinkish). Despite these variations, the colorimetric readouts remained consistent, which can be attributed to the optimized methylene blue concentration in the dye pad. More importantly, the electrochemical signals were robust and comparable across all sample types, underscoring the assay’s reliability and suitability for application in complex biofluids. In addition, the target in our study is a ssDNA sequence. Hence, if the assay were to be utilized for double-stranded DNA (dsDNA), it would be essential to first denature the dsDNA into ssDNA before analysis. Another area of investigation would be to explore the specificity with other combinations of mismatches, as the position of mismatched bases may affect hybridization and signal. Furthermore, careful considerations must be taken while designing the assay for target and nonspecific sequences with higher G content, as it may generate larger specific and nonspecific signals due to increased G base electrostatic quenching with MB. Nevertheless, in the future, this assay can easily be reprogrammed for any ssDNA (as is) or ds DNA (post DNA lysis) “Disease X” pathogen and validated in 3–5 months, expediting the technology development cycle for rapid detection and outbreak mitigation.

## Methods

### Materials

The deposition Buffer used in the experiments consisted of a 1x buffer, 10 mm MgCl_2_, 0.05% Tween 20 (polyoxyethylene sorbitan monolaurate) from IDT and Sigma. The running buffer (1xssc- Sodium Saline Citrate), Methylene blue, Grade 1 chromatography paper, and DNA oligonucleotides were purchased from Sigma, Thermo Fisher Scientific, Whatman, and IDT, respectively. Agarose was procured from Sigma, 10x Tris Boric acid EDTA buffer from Biorad, GeneRuler Ultra Low Range DNA Ladder (10–300 bp), DNA Gel Loading Dye (6x) from Thermofisher Scientific and GelRed stain from Sigma. The sequences of ssdna oligos can be found in Table S1(Supplementary Information). Pooled human AB serum (plasma-derived heat-inactivated), single donor human plasma (blood-derived), and single donor human whole blood were all collected from Innovative Research Inc. The ABS Pro filament required to manufacture the casing was purchased from Filaments.ca. Ag/AgCl paste was purchased from Sigma and used as a reference electrode.

### MebiQue-LFA fabrication, assembly, and optimization

For the assay, a smooth finished chromatography paper (CHR, Grade 1), 0.18 mm thick with a linear flow rate (water) of 130 mm/30 min, was used due to its consistent performance, excellent wicking properties, and suitability for facilitating capillary flow in diagnostic applications^48,49^. This paper was manually sliced into slender pieces, each measuring 6 cm long and 0.5 cm wide. The strip featured components such as a 1 cm sample pad (where the target is added), a 1 cm conjugation pad (where the target conjugates with reporter probe), a 1 cm dye pad (signal tracer- methylene blue), a 0.2 cm test line (capture probe), and 1.8 cm wicking pad (where the sample is wicked to the end of the test strip). The 1 cm x 0.5 cm dye pad assembly involved depositing 10 µL of 3.5 mM of methylene blue. The test line was prepared by drawing a line with 0.2 cm width using a 3D printed dispenser. The Test line capture probe was diluted to a concentration of 110 µM in the deposition buffer, and using the dispensing mechanism, a volume of 10 µL was added to the test line. For the conjugation pad measuring 1 cm x 0.5 cm, the conjugate probe was diluted to 5 µM in deposition buffer, and a volume of 10 µL was added to the pad.

The Target analyte, a synthetic ssDNA oligonucleotide, was diluted to a concentration of 1 µM in the running buffer (1xSSC), and a volume of 30 µL was utilized for experimental procedures. In contrast, for the control, only the running buffer was employed to carry out the experiments. After the assays’ preparation, they were heated on a hot plate at 60֯C for 5–10 min. Subsequently, the assays were allowed to dry at room temperature once more for 1–2 h and stored until use. Chromatography paper strips were precisely cut to a length of 6 cm, with widths varying from 0.5 cm to 2.5 cm. The analyte utilized in these experiments consisted of a solution of blue food dye, with a consistent application volume of 100 µL across all trials. The duration it took for the analyte to reach the test line was recorded, aiming to identify the most effective width for the assay.

An additional supplementary test was conducted to determine the optimal volume for the assay, maintaining consistent dimensions for the strips while varying the volume within the range of 15 µL to 75 µL. Similarly, a blue food dye solution was served as the analyte, and the optimal volume was based on the duration required for the solution to reach the test line. Finally, the strip with all pads was loaded with analyte, excluding the conjugate pad, and was tested with 30 µL of target DNA over 9 min to assess test line visibility.

The cartridge design was created using Fusion360 CAD modeling software and 3D printed using a QIDI X-PLUS II Printer and ABS pro filament. The choice of ABS filament ensured robustness and durability, essential for the cartridge’s reliability in experimental conditions. The slice specifications used to 3D print ABS filament can be found in Table S2.

The PCB electrodes were designed using KiCAD software and manufactured using conventional ENIG plating (PCBway.com). The electrodes’ redox behaviours were investigated using 0.3-3 mM methylene blue and 2 mM [Fe(CN)6]^4−^/[Fe(CN)6]^3−^ solutions against Ag/AgCl. Ag/AgCl reference electrodes were prepared by applying a thin layer of Ag/AgCl paste onto the reference electrodes, followed by drying at 60 °C for 10 min. In the methylene blue analysis, solutions of 0.3 mM and 3.0 mM methylene blue in DI water were prepared. 30 µL of these solutions were individually deposited onto the electrodes, and square wave voltammetry was performed from 0 to -0.6 V. On the other hand, cyclic voltammetry (CV) was performed using 30 µL of 2 mM [Fe(CN)6]^4−^/[Fe(CN)6]^3−^ in 25:25 buffer containing 25 mM phosphate buffer saline (PBS) and 25 mM NaCl. The CV scans were obtained from − 0.5 to 0.5 V, with 0.01 V steps, and at various scan rates (25 mV/s, 50 mV/s, and 100 mV/s).

### Strand hybridization assay validation using gel electrophoresis

To determine the interaction of DNA i.e. ssDNA (Target analyte probe (TP), Conjugation Pad probe (CP) and Test Line (TL)) and dsDNA (TP + CP and TP + CP + TL) agarose gel electrophoresis (AGE) was conducted. The samples (a) TP + CP (b) TP + CP + TL were incubated in TE buffer 20 µL (total volume 20 µL, final DNA concentration 1 µM each) at 70˚C for 60 min before loading the reaction mixture on a 5% agarose gel stained with GelRed staining dye. The gels were run at 50 V for 3 h in 1x Tris base/boric acid/ethylenediaminetetraacetate (TBE) buffer (ph 8.3) and visualized after UV excitation on a BioRad ChemiDoc touch imaging system. Although the target and CP tend to undergo self-dimerization, a significantly high self-dimerization signal on agarose gel differs from the actual linear form. Despite the self-dimerization tendency of both the Target and CP, the linear DNA signal and the interaction remain distinct on the gel.

### Analytical performance validation of MebiQue-LFA

The assay’s analytical performance underwent evaluation through two key experiments: sensitivity and specificity assessments. The objective of the sensitivity test was to observe the assay’s reaction and response across varying concentrations of the test probe. Seven different concentrations, ranging from 0 to 1000 nM (with the lowest concentration of the target being 1 pM) were employed in this experiment. The prepared assays systematically introduced the six varying target concentrations (30 µL) and a control (no target). The corresponding signals at the test line were measured by the electrode using Sensit Smart (square wave voltammetry, 0 to -0.3 V) and visually. The experimental limit of detection (LOD) was determined visually, and the calculated LOD was evaluated by substituting the limit-of-blank as the “y” value in the regression line equation of the calibration curve. Limit of blank (LOB) for LOD for spiked ssDNA in blood: *LOB = mean of blank (blood) + 3×( S.D.of blank)*. Since the x-axis is on a logarithmic scale, we applied the antilog to convert the value from the log scale to a linear scale.

Concurrently, four nonspecific mismatch scenarios, namely 1 bp, 2 bp, 3 bp mismatches, and complete non-complementary targets, were introduced to gauge the assay’s selectivity and specificity. Similar to the sensitivity test, the assay included the specified concentrations and volumes in each pad and a 30 µL volume 1 µM of mismatches; 1base mismatch: 1MM, 2bases mismatch: 2MM, 3bases mismatch: 3MM, Noncomplementary: NC and 100 pM of specific target was used for this test. ImageJ software v1.54 was employed to analyze the mean light intensity of the development of the colour in the testing region during experiments. To compare the ssDNA and dsDNA targets study, 30 µL of 1 µM of CP and TP + CP was run on the lateral flow assay, and the signals were measured visually. Visible spectroscopy measurement was performed on 50 µM methylene blue (MB) and MB (50 µM) spiked TP (100 nM), CP (100 nM), mismatched sequences (100 nM:1MM and 2MM), TP (100 pM, 1nM and 100 nM) + CP (100 nM). The samples were incubated for 1 h at 4$\:℃$. The absorbance measurements were performed using Nanodrop Onec (Thermo Fisher Scientific) in the 450–700 nm wavelength range.

A comprehensive stability test was performed to assess the long-term stability and reliability of the lateral flow assay. The test aimed to determine the assay’s performance over time when stored under different environmental conditions. LFS were prepared in triplicate and stored for 3,5, and 10 days at two distinct conditions: ambient room temperature and refrigerated storage at 4$\:℃$. These conditions were chosen to simulate typical storage environments that the assay might encounter in real-world applications. The Coefficient of Variation % was calculated using the SD/mean× 100 formula. The test strips were prepared using the concentrations and volumes specified in the material section, ensuring consistency across all trials. At each designated time point and storage condition, 30 µL of Target DNA at a concentration of 1 µM was applied to the strips for the experiment. Then, the electrochemical response of each strip was subsequently analyzed using square wave voltammetry (SWV).

Human plasma, serum, and whole blood were employed in the experiment, incorporating six distinct concentrations ranging from 0 nM to 100 nM. A 10 µM solution of the target was diluted in a running buffer to achieve the desired concentrations. It was serially diluted with plasma, serum, or whole blood in vials to create the different test dilutions.

To optimize the assay’s performance in contrived whole blood, we executed a series of dilution tests encompassing concentrations ranging from the lowest, 0 nM, to the highest, 100 nM. For the 0 nM concentration, representing whole blood without the target probe, meticulous dilutions of 10% (lowest blood concentration), 30%, and 50% (highest blood concentration) in saline-sodium citrate (SSC) buffer were conducted. Conversely, for the highest concentration of 100 nM, 10 µM of the target was serially diluted in 10%, 30%, and 50% of the whole blood. Furthermore, the different concentrations of the targets spiked in blood and diluted to 50% were tested. During testing, signals at the test line were detected by the electrode, and data collection was performed using Sensit Smart (square wave voltammetry, 0 to -0.3 V), which was connected to a smartphone visually. The fold increase values were obtained using the standard formula.

## Supplementary Information

Below is the link to the electronic supplementary material.

Supplementary Material 1

## Data Availability

The dataset generated and/or analyzed during the current study are available from the corresponding author on reasonable request.
