# Supplementary material for: Methylene blue as a new signal tracer for nucleic acid-based lateral flow assay
Source: Sci Rep. 2025 Oct 14;15:35900. doi: 10.1038/s41598-025-19701-4 (PMC12521506; doi:10.1038/s41598-025-19701-4)
Supplement: Supplementary file 1 — Supplementary Material 1 [file 41598_2025_19701_MOESM1_ESM.docx]

Methylene Blue as a new signal tracer for Nucleic Acid-Based Lateral

Flow Assay

Jurjaan Onayza Noim^a*^, Dhruvi Kakadiya^a^, Stephanie Dang^a^, Nabil Royez^b^, Shruti Ahuja^c^, Krishna Prasad Aryal^a^, Siddharth Tallur^d^, Dylan Ravindran Pillai^b,e^, Richa Pandey^a,f*^

*^a^Department of Biomedical Engineering, University of Calgary*

*^b^Department of Microbiology, Immunology, and Infectious Diseases, University of Calgary*

*^c^Centre for Research in Nanotechnology & Science (CRNTS), Indian Institute of Technology (IIT) Bombay*

*^d^Department of Electrical Engineering, Indian Institute of Technology (IIT)*

*^e^Department of Pathology and Laboratory Medicine, University of Calgary*

*^f^Hotchkiss Brain Institute, University of Calgary*

^*^Corresponding Author

Richa Pandey - Department of Biomedical Engineering, University of Calgary, (Canada) 2500 University Dr NW, Calgary, AB T2N 1N4, Hotchkiss Brain Institute, University of Calgary (Canada) 3330 Hospital Dr NW, Calgary, AB T2N 4N

Email: [richa.pandey@ucalgary.ca](mailto:richa.pandey@ucalgary.ca)

**Supplementary Methods:**

Casing fabrication

The cartridge design was meticulously created using Fusion360 CAD modeling software. After finalizing the CAD model of the cartridge, it was 3D printed using a QIDI X-PLUS II Printer and an ABS pro filament. The choice of ABS filament ensured robustness and durability, essential for the reliability of the cartridge in experimental conditions. The slice specifications used to 3D print ABS filament can be found in Table S2.

Lateral flow assay strip optimization

Chromatography paper strips were precisely cut to a length of 6 cm, with widths varying from 0.5 cm to 2.5 cm. The analyte utilized in these experiments consisted of a solution of blue food dye, with a consistent application volume of 100 μL across all trials. Meticulously, the duration it took for the analyte to reach the test line was recorded, aiming to identify the most effective width for the assay.

 To determine the optimal volume for the assay, a supplementary test was performed, maintaining consistent dimensions for the strips while manipulating the volume within the range of 15 μL to 75 μL. It is important to note that this experiment was conducted without a complete assay setup and did not include a test line. The primary objective was to evaluate the flow dynamics by measuring the time required for different sample volumes to traverse a single-length lateral flow strip. This assessment focused exclusively on flow rates and was independent of any target-analyte interactions or signal generation. Similar to the previous experiment, a blue food dye solution was served as the analyte. The selection of the optimal volume was based on the duration required for the solution to reach the test line.

Subsequently, for other experiments, the strip was subjected to an additional test wherein all pads were loaded with the requisite amount of analyte, excluding the conjugate pad, which remained devoid of DNA. Following this setup, a standard testing procedure was followed using 30 μL of target DNA, and the strip was observed for 9 minutes to assess the presence of any visible test line.

Spectroscopy measurement

Visible spectroscopy measurement was performed on 50 μM methylene blue (MB) and MB (50 μM) spiked TP (100 nM), CP (100 nM), mismatched sequences (100 nM:1MM and 2MM), TP (100 pM, 1nM and 100 nM) + CP (100 nM). The samples were incubated for 1 hour at 4֯C and the absorbance measurements were performed using Nanodrop Onec (Thermo Scientific™) for a wavelength range of 450- 700 nm.

**Supplementary Tables**

**Table S1.** This table presents crucial ssDNA sequences designed for various assay components. The same color texts in the sequences, such as black, red and blue, denote complementarity. Grey highlights denote the mismatch.

| Name | Length | Sequences |
| --- | --- | --- |
| Target analyte | 68 | 5’ TTT CTT GGA TGG TGA TGC ATG GCC GTT TTT AGT TCG TGA ATA TCG TAT TTG CCG CTA ATT AGC AGG TT 3’ |
| Conjugation pad | 86 | 5’ AA CCT GCT AAT TAG CGG CAA ATA CGA TAT TCA CGA ACT AAA AAC GGC CAT GCA TCA CCA TCC AAG AA AAA AAA AAA AAA AAA AAA 3’ |
| Test Line | 20 | 5’ TTT TTT TTT TTT TTT TTT TT 3’ |
| 1 base pair mismatch | 68 | 5’ TTT CTT GGA TGG TGA TGC ATG GCC GTT TTT AGT TCG TGA ATA TCG TAT TTG CCG CAA ATT AGC AGG TT 3’ |
| 2 base pair mismatches | 68 | 5’ TTT CTT GGA TGG TGA TGC ATG GCC GTT TTT AGT TCG TGA ATA TCG TAT TTG GCG CAA ATT AGC AGG TT 3’ |
| 3 base pair mismatches | 68 | 5’ TTT CTT GGA TGG TGA TGC ATG GCC GTT TTT AGT TCG TGA ATA TCG TAT TTG GCG CAA ATT AGC AGT TT 3’ |
| Non-complementary | 68 | 5’ AAA GAA CCT ACA ACA ACG AAC GGG CAA AAA TCA AGC ACA TAT AGC ATA AAC CGC GTT TAA ATG TCA AA 3’ |

**Table S2.** This table provides the slice specifications and printing parameters for 3D printing ABS filament to create the cartridge. These parameters collectively define the conditions under which ABS filament is processed to ensure accurate and optimal printing results for the cartridge components.

| Slice Specifications and printing parameters [ ABS Material] | |
| --- | --- |
| Nozzle Temperature | 250°C |
| Print Speed | 60 mm/s |
| Build Plate Temperature | 100 |
| Chamber Temperature | 45°C |
| Infil Density | 25 % |
| Printing Temperature | 240°C |
| Travel Speed | 100 mm/s |

**Table S3.** This table reports the stability of Assay Performance at Different Storage Conditions Over 10 Days for a 1 µM Target Concentration.

|  | Room Temperature | | 4 ֯ C | |
| --- | --- | --- | --- | --- |
| Days | Mean$\boldsymbol{\pm}$ SD | CV% | Mean$\boldsymbol{\pm}$ SD | CV% |
| 0 | 1.34$\pm$0.15 | 11.20 | 1.34$\pm$0.15 | 11.20 |
| 3 | 1.18$\pm$0.45 | 38.40 | 1.17$\pm$0.45 | 38.06 |
| 5 | 1.17$\pm$0.32 | 27.40 | 1.53$\pm$0.40 | 26.36 |
| 10 | 1.40$\pm$0.44 | 31.13 | 1.43$\pm$0.38 | 26.41 |

**Supplementary Figures**


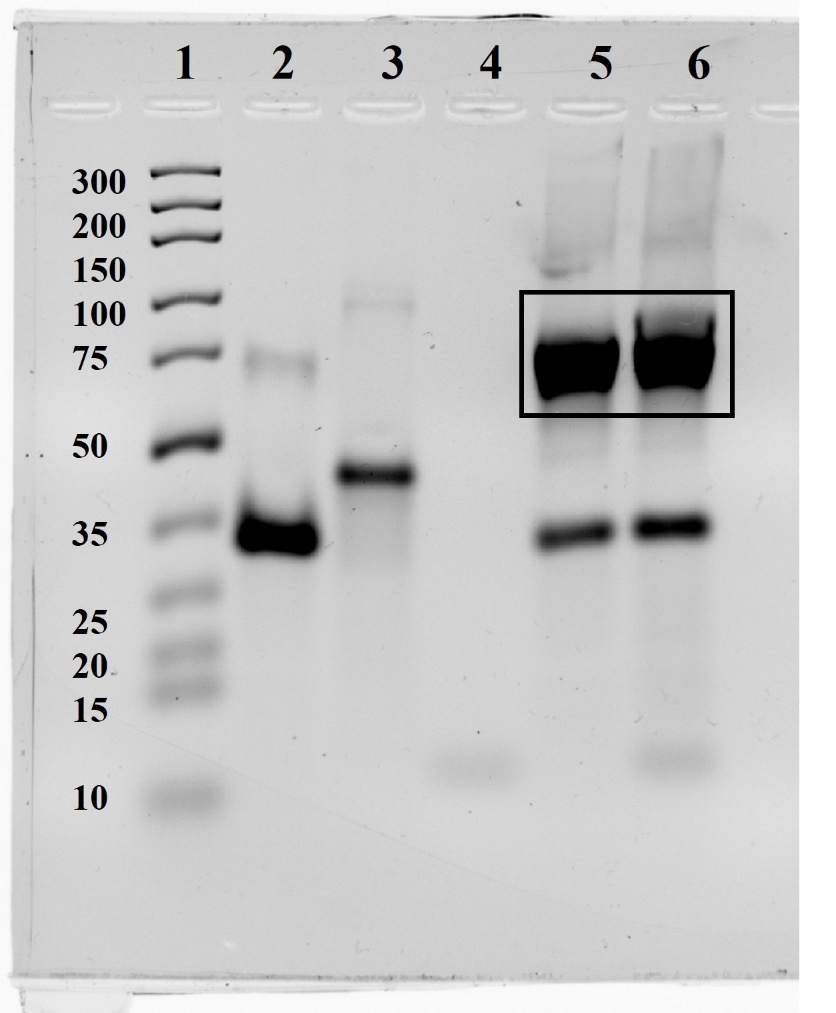


**Figure S1.** Gel study of the designed assay in solution. Interaction of different sequences monitored by electrophoretic mobility in 5% agarose gel. Lane 1 = 10-300 bp DNA ladder marker (Thermofisher); lane 2 = Target analyte (TP: 68 nt); lane 3 = Conjugation pad (CP: 86nt); lane 4 = Test line (TL: 20 nt); lane 5 = TP+CP (68 bp); lane 6 = TP+CP+TL (85 bp). The highlighted bands show the shift observed due to the interaction between TP+CP (lane 5) and TP+CP+TL (lane 6). The target and CP typically experience self-dimerization, leading to a notably strong self-dimerization signal on agarose gel (lane 2,3,5 and 6), this signal appears distinct from the linear form.


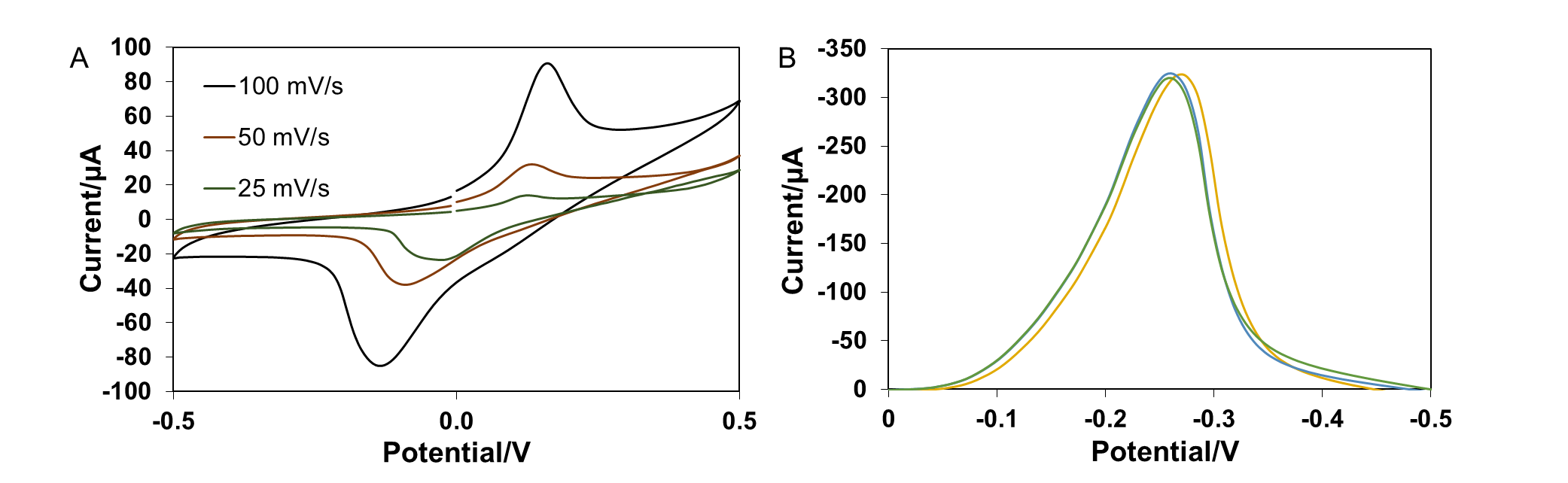


**Figure S2.** PCB electrodes characterization A) Cyclic voltammetry (CV) of 2 mM [Fe(CN)6]^4-^/[Fe(CN)6]^3-^ against Ag/AgCl at 100 mV/s, 50 mV/s, and 25 mV/s. B) Square wave voltammetry of 3.5 mM methylene blue solution against Ag/AgCl.


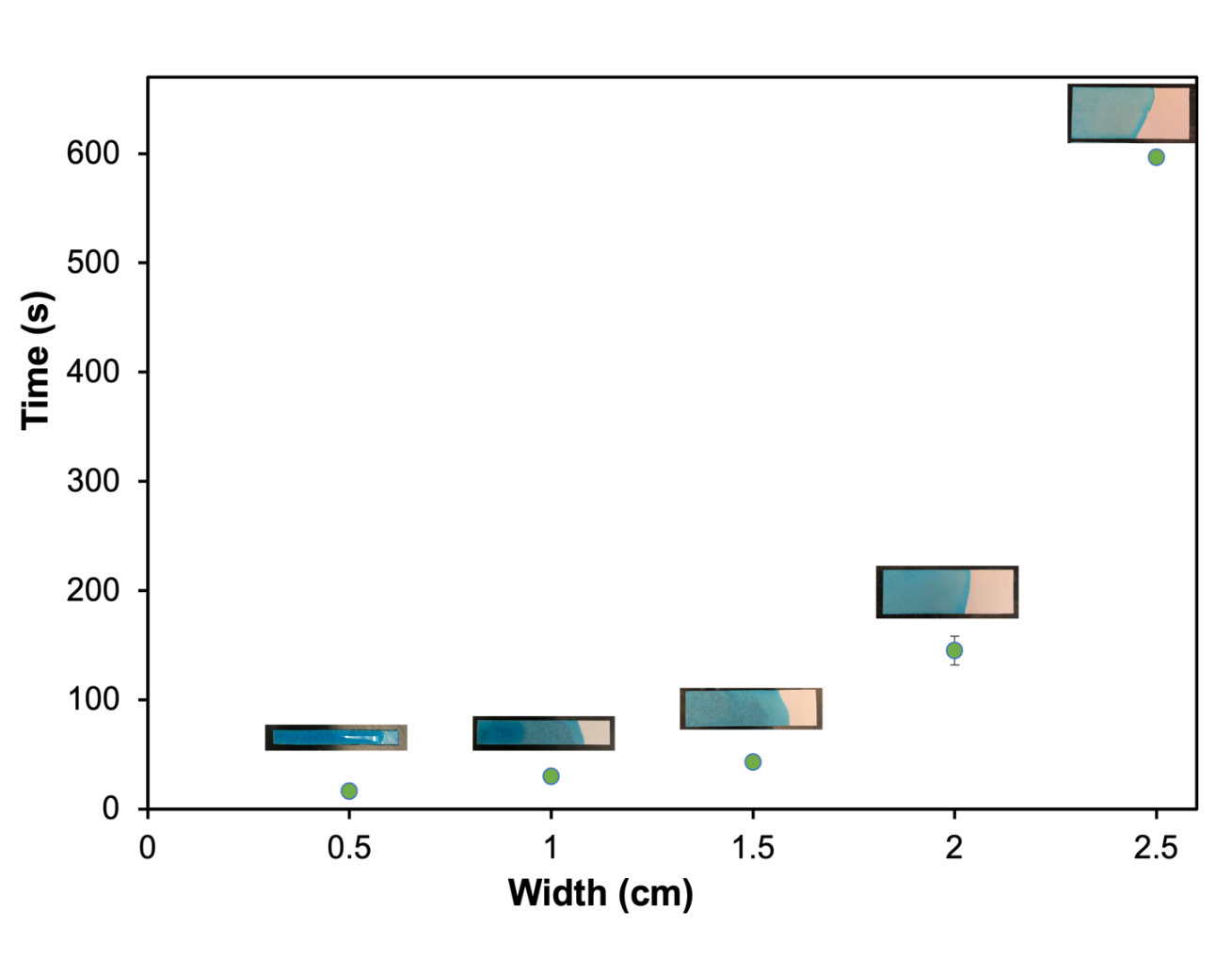


**Figure S3.** Lateral flow strips with varying widths (0.5, 1, 1.5, 2, and 2.5 cm) alongside average time data points illustrate the correlation between strip width (cm) and the corresponding fluid flow time (s). The error bars represent the standard deviation of three devices. The pictures show the visual representation of the corresponding fluid flow.


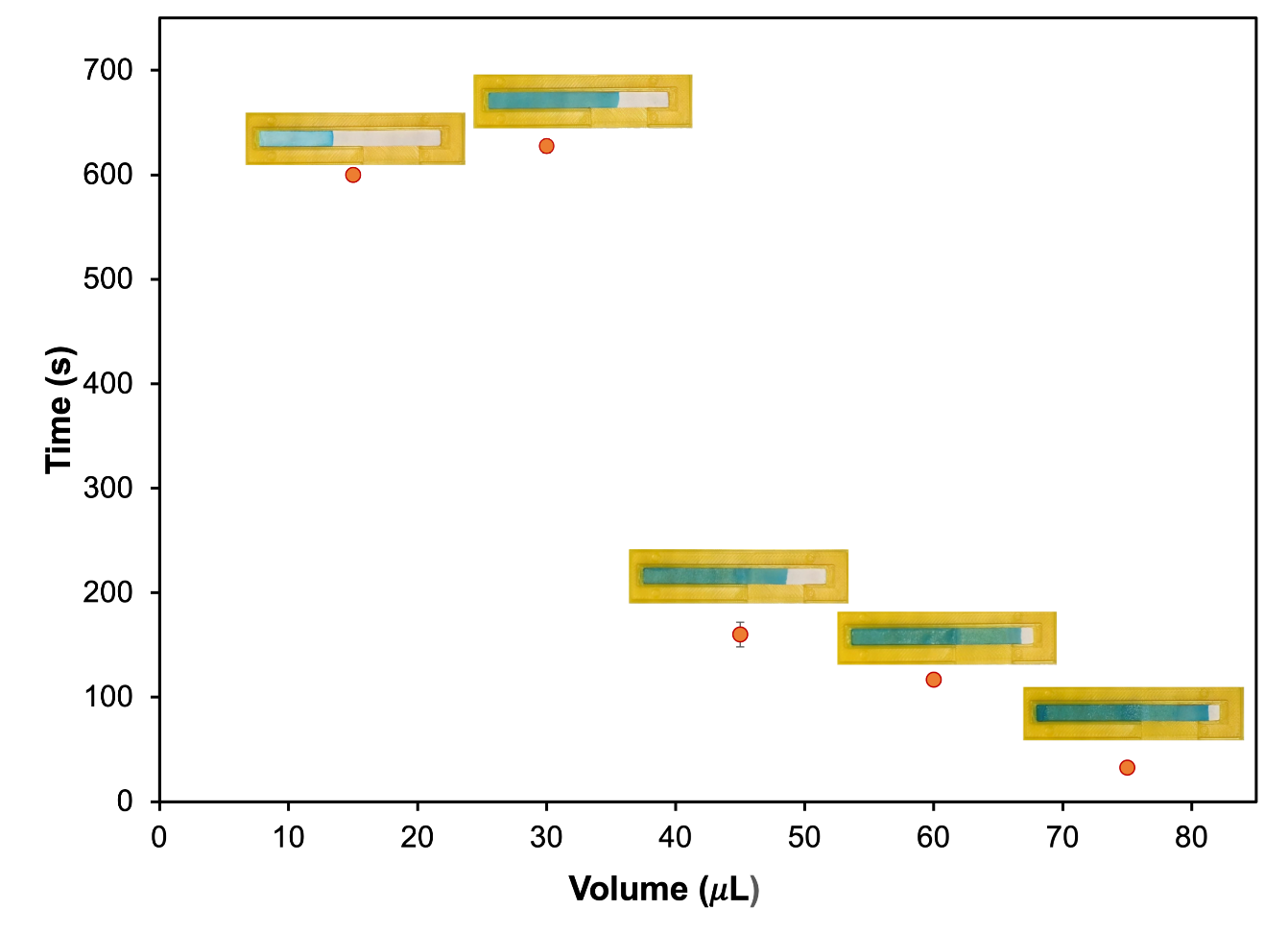


**Figure S4.** Lateral flow strips set at a consistent width of 0.5 cm, were tested with different fluid volumes (15, 30, 45, 60, and 75 μL). The corresponding plot delineates the correlation between distinct volumes (μL) and the average of three devices. The pictures show the visual representation of the corresponding fluid flow.


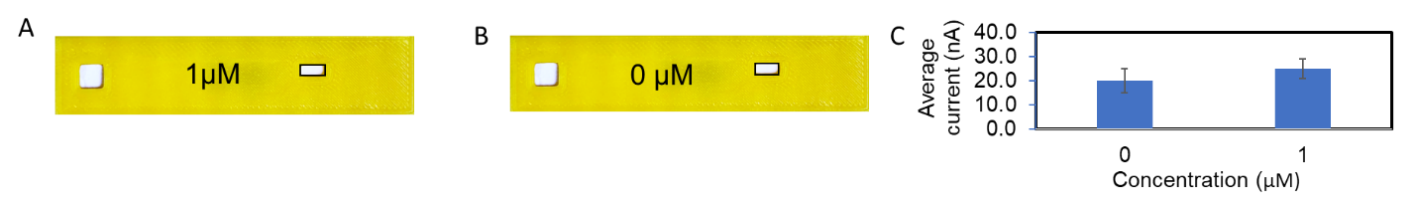


**Figure S5.** A) LFA performance to show 1μM ssDNA (no CP sequence) interaction with the MB to generate the test line. B) LFA performance to show 0μM ssDNA (control) interaction with the MB to generate the test line. The study was conducted in three devices, with one device serving as the test and the other as the control, and panels A and B representing each device, respectively. The target used in this study is a single-stranded synthetic oligonucleotide (ssDNA), and no CP sequence is used; hence, the ssDNA is supposed not to interact with the MB in the dye pad to generate any signal. C) Square wave voltammetry measurement of the three devices demonstrating average current vs concentration. The error bar indicates the standard deviation of n=3 measurements.

**
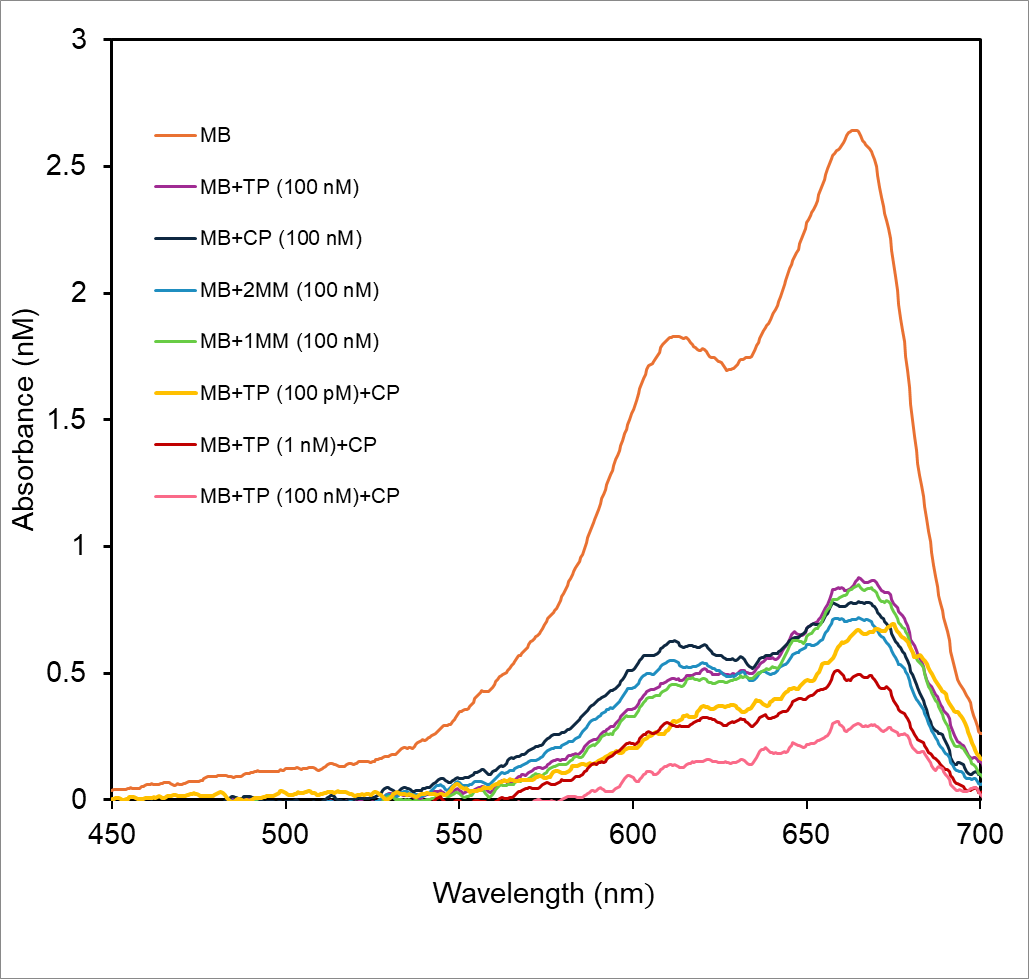
**

**Figure S6.** Visible spectroscopy study of the methylene blue (MB), and target probe (TP), conjugation probe (CP) sequences, mismatches (1MM and 2MM) in the presence of MB.


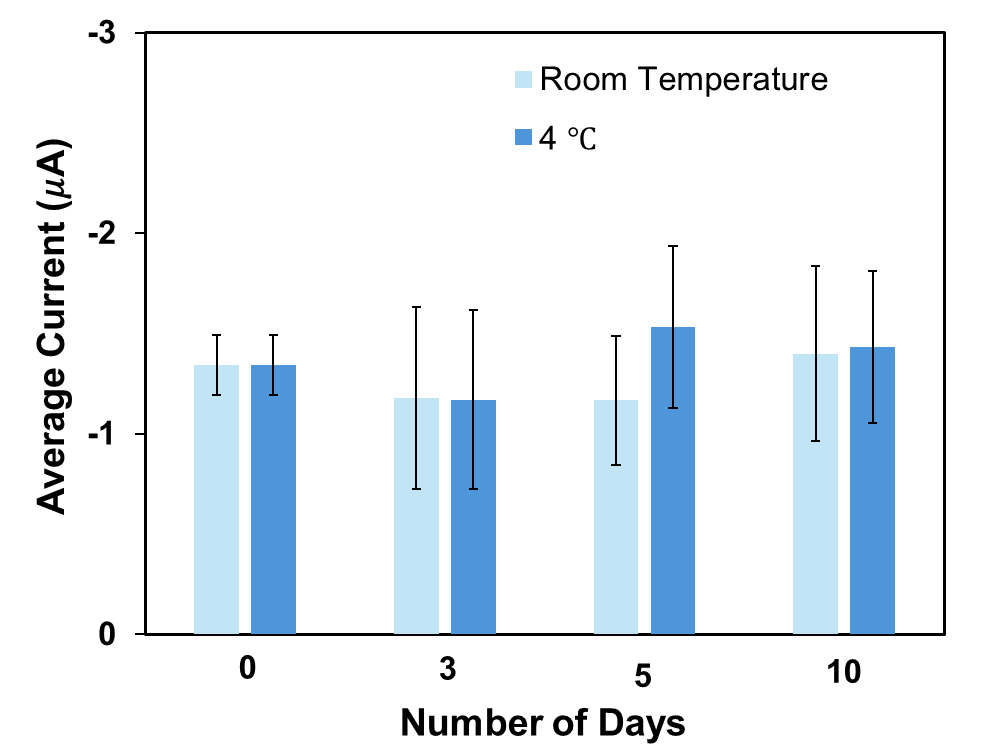


**Figure S7:** Bar plot illustrates the stability test results for the assay, conducted over a 10-day period at room temperature and 4°C. The plot depicts the relationship between the number of storage days and the average current measured, providing a comparative analysis of stability under the two conditions.


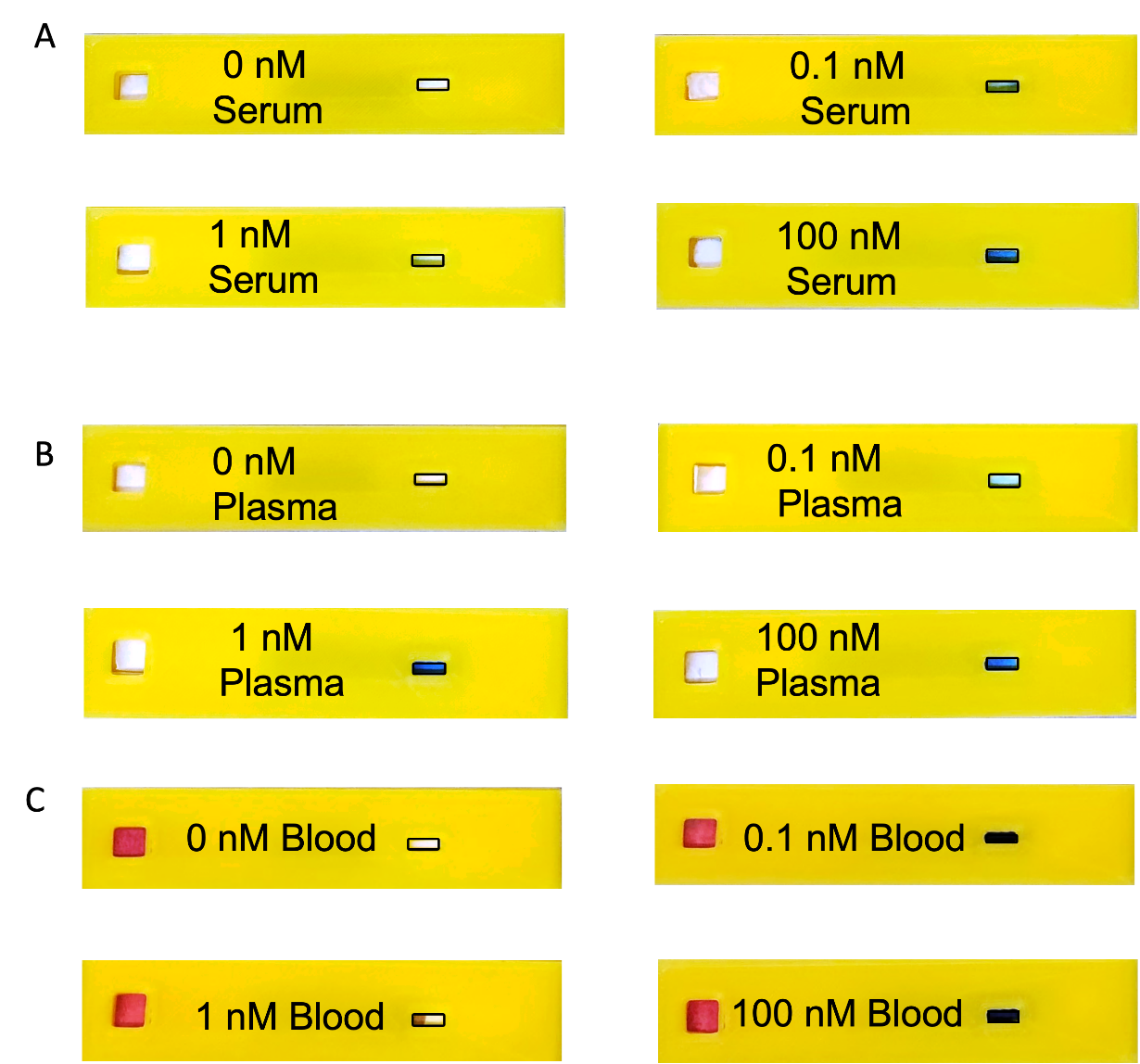


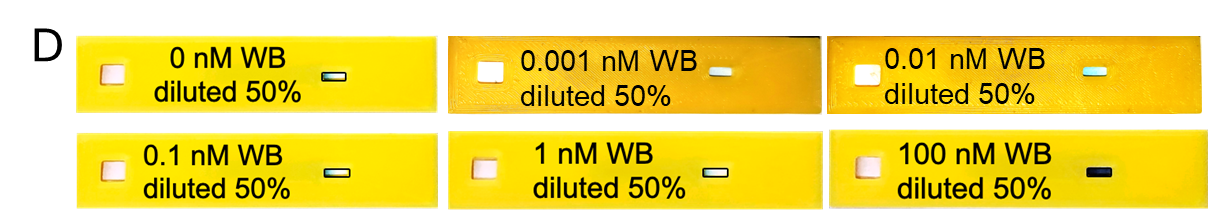


**Figure S8.** Visual representation illustrating the performance of the LFA using A) human serum, B) human plasma, C) whole blood, D) Diluted 50% whole blood. All the measurements were done across a range of target DNA concentrations from 0nM to 100nM.


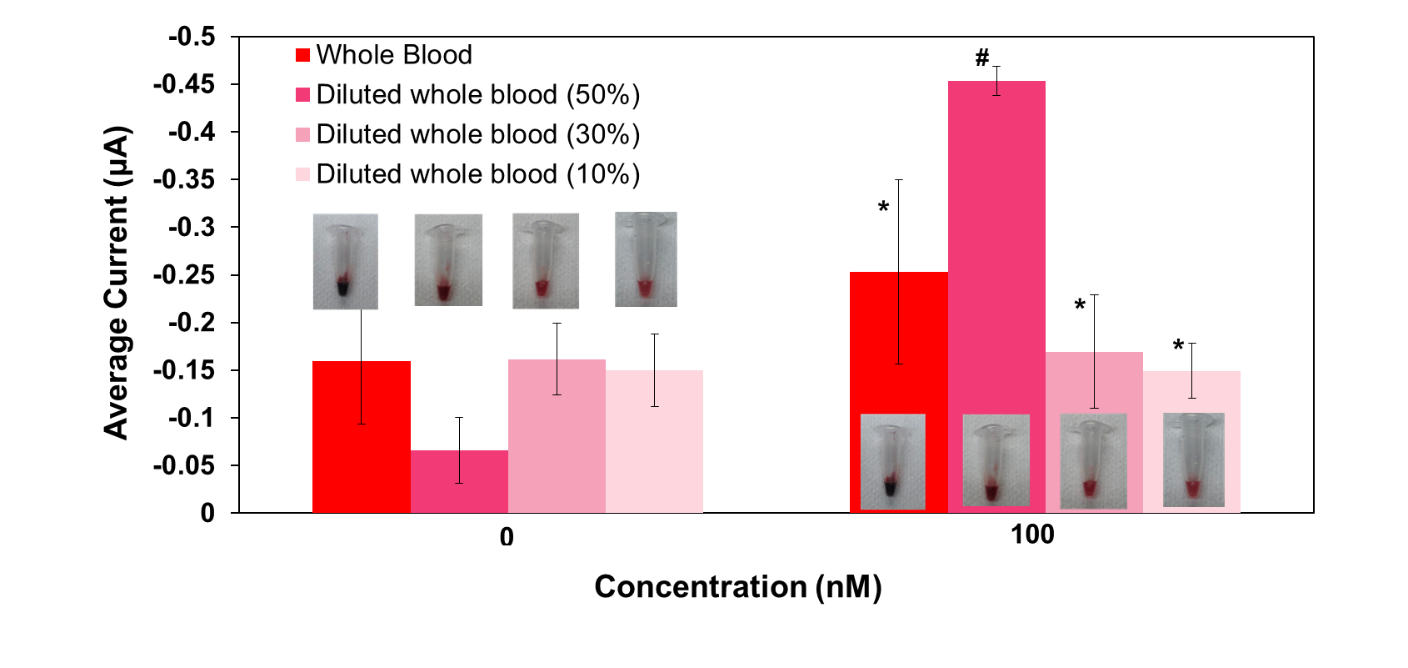


**Figure S9.** Visual representation of whole blood dilution (10%, 30%, and 50 %) spiked with 0nM and 100nM of target DNA concentrations. Bar chart representing average currents generated for concentrations of 0nM and 100nM target DNA with corresponding error bars. Three devices were tested. Here p value * >0.05 and # <0.05.
